# Supplementary material for: Developing guidelines for culturally relevant informed consent: an example from Lebanon
Source: PLOS Ment Health. 2025 Apr 17;2(4):e0000174. doi: 10.1371/journal.pmen.0000174 (PMC12798495; doi:10.1371/journal.pmen.0000174)
Supplement: S2 Checklist — (DOCX) [file pmen.0000174.s002.docx]

**Checklist for Culturally Relevant Informed Consent**

This checklist is grounded in the findings of the research and the accompanying manual, which was designed to provide a comprehensive framework for ensuring that informed consent processes are culturally sensitive, inclusive, and responsive to the needs of participants. It serves as a practical tool for researchers to facilitate these objectives in their work.

In addition, the checklist emphasizes that informed consent is not a one-time intervention but an ongoing process throughout the entire research journey. As you use it, please keep in mind that informed consent should be revisited and maintained regularly, ensuring it evolves in a way that respects participants' autonomy, cultural context, and evolving needs over time.

1. Pre-Consent Preparation

- Conduct a diagnostic survey to assess current consent practices and areas for improvement.
- Engage Community Advisory Boards (CABs) early to co-develop consent materials and strategies.
- Identify participant values to address potential gaps in consent content.
- Evaluate power dynamics between researchers and participants to mitigate coercion or undue influence.

2. Consent Materials & Delivery

- Co-design consent forms and materials with CABs to ensure clarity and cultural relevance.
- Ensure consent documents are available in local languages and tailored to literacy levels.
- Offer multiple formats (written, verbal, visual, or digital) to accommodate diverse preferences.
- Clearly outline research benefits, risks, time commitments, and withdrawal rights in an accessible way.
- Avoid lengthy, overwhelming documents by using summarization techniques and supplementing with discussions.

3. Consent Process & Participant Interaction

- Use role-play scenarios with researchers to practice and refine culturally appropriate consent approaches.
- Conduct group information sessions followed by individual conversations to enhance comprehension.
- Ensure researchers are warm, approachable, and professional while respecting boundaries.
- Implement Common Problems Cards for reflective discussions on potential participant concerns.
- Confirm understanding by having participants rephrase key points in their own words.
- Provide clear ongoing consent mechanisms, allowing participants to reconsider or ask questions anytime.

4. Ethical Considerations & Community Involvement

- Train researchers in cultural competence, reflexive questioning, and ethical rapport-building.
- Recruit community members as outreach staff, and researchers when applicable, to enhance trust and accessibility.
- Create a wish list from CAB and participant input to adapt consent practices to community needs.
- Address social and power dynamics, ensuring participation is voluntary and free of coercion.

5. Continuous Improvement & Monitoring

- Gather participant feedback and adjust consent practices accordingly.
- Conduct ethics audits to evaluate adherence to culturally sensitive consent processes.
- Encourage co-learning between researchers, CABs, and participants to refine practices over time.
- Regularly assess barriers to participation (gender norms, logistical challenges, societal influences) and adapt strategies.
