# Supplementary material for: Developing guidelines for culturally relevant informed consent: an example from Lebanon
Source: PLOS Ment Health. 2025 Apr 17;2(4):e0000174. doi: 10.1371/journal.pmen.0000174 (PMC12798495; doi:10.1371/journal.pmen.0000174)
Supplement: S3 Checklist — (DOCX) [file pmen.0000174.s003.docx]

Inclusivity in global research

PLOS’ policy on inclusivity in global research aims to improve transparency in the reporting of research performed outside of researchers’ own country or community and ensures that PLOS publications reporting global research adhere to high standards for research ethics and authorship. Authors of relevant research articles may be asked to complete the questionnaire below, which outlines ethical, cultural, and scientific considerations specific to inclusivity in global research. This questionnaire may be requested when researchers have travelled to a different country to conduct research, if research uses samples collected in another country, research with Indigenous populations or their lands, or if research is on cultural artefacts. Researchers travelling to another country solely to use laboratory equipment will not normally be required to complete the questionnaire. However, the questionnaire can be requested at the journal’s discretion for any submission – if you have been requested to complete this questionnaire by the PLOS journal you submitted to, please do so.

Please complete the questionnaire below and include this as a Supporting Information file with your manuscript. Note that if your paper is accepted for publication, this checklist will be published with your article in the supporting information files. Please ensure that you reference the checklist in the main body of your manuscript. We suggest adding a subsection ‘Inclusivity in global research’ to your Methods section and adding the following sentence: “Additional information regarding the ethical, cultural, and scientific considerations specific to inclusivity in global research is included in the Supporting Information (SX Checklist)”

The questions have been designed to be applicable to a wide range of study types, and there are subsections for both human subjects research and non-human subjects research. If any of the questions are not relevant to your research please mark them as “N/A” as appropriate.

**Ethical considerations, permits and authorship**

*This section is applicable to all research types.*

**Provide details as to who granted permissions and/or consent for the study to take place in the Methods section of your manuscript. This should include the names of all ethics boards, governmental organizations, community leaders or other bodies that provided approval for the study. If individuals provided approval refer to these people by their role or title but do not list their name(s).**

Reported on page number: 13

**If there were any deviations from the study protocol after approval was obtained please provide details of these changes in the Methods section of your manuscript.** **Did this study involve local collaborators that are residents of the country where the research was conducted or members of the community studied? If you do not have any authors from said communities, please provide an explanation for this below.**

Reported on page number:

N/A

*Yes, this study involved collaboration with members of the community being studied, the Community Advisory Board mentioned in the methodology section, p.7-8. From the outset, we aimed to include local collaborators as authors to ensure their perspectives were adequately represented. However, as the project neared the prototyping stages, the war in Lebanon disrupted our ability to travel and engage directly with these collaborators during the final stages of the research.*

*We explored the possibility of involving them remotely by having them review the manuscript and provide feedback. However, this would have required translating the document into Arabic due to language differences, which introduced additional time and budgetary constraints. Given that the project and its funding were nearing completion, we regrettably could not accomplish this step.*

*While these challenges were beyond our control, we remain committed to principles of inclusive and community-driven research. In future projects, we will proactively consider contingencies for unforeseen circumstances to better uphold this commitment.*

Everyone listed as an author should meet PLOS’ criteria for authorship and all individuals who meet these criteria should be included in the author byline, rather than the acknowledgements. For further information please see the journal’s Authorship Policy.

**Human subjects research (e.g. health research, medical research, cross-cultural psychology)**

**Did you obtain written informed consent from a representative of the local community or region before the research took place? How did you establish who speaks for the community?** **Details of written informed consent obtained from study participants should be reported separately in the Methods section of your manuscript.**

*We did not obtain written informed consent from a specific representative of the local community. As detailed in the Methodology section (pages 7–8), members of the community were invited to participate through their existing involvement in the Community Advisory Board (CAB) developed for a prior large-scale research project at War Child that focused on designing and evaluating a psychological intervention for vulnerable families. The CAB had previously collaborated closely with one of the researchers on the War Child team, establishing a strong rapport. This existing relationship made this researcher the focal point for engaging with the community.*

*Details of the written informed consent obtained from study participants are reported separately in the Methods section (page 13).*

**How did members of the local community provide input on the aims of the research investigation, its methodology, and its anticipated outcome(s)?**

*Members of the local community provided input on the aims of the research investigation, its methodology, and its anticipated outcomes through several steps. First, participants were clearly informed about the details of the project via the Participant Information Sheet. As described in the Methodology section (page 8), the steps of the project were communicated to participants to ensure transparency. Additionally, to determine the desired level of participation of the Community Advisory Board (CAB) members and NGO staff in this study, ELRHA’s Participation in Humanitarian Innovation Toolkit was utilized, facilitating a structured and inclusive approach to collaboration.*

**When engaging with the local community, how did you ensure that the informed consent documents and other materials could be understood by local stakeholders?**

*When engaging with the local community, we ensured that the informed consent documents and other materials were accessible and understandable by thoroughly translating them into Arabic. These documents were then read aloud and discussed in a group setting with the participants to ensure clarity and address any questions or concerns before obtaining their approval to join the research.*

**Will the findings of the research be made available in an understandable format to stakeholders in the community where the study was conducted (e.g. via a presentation, summary report, copies of publications, etc.)? Please provide details of how this will be achieved.**

*Yes, the findings of the research will be made available in an understandable format to stakeholders in the community where the study was conducted. As part of the broader GOAL project, communication materials were developed in English and then translated into Arabic to ensure accessibility for community stakeholders. Additionally, throughout the research, we shared results and analyses in Arabic to keep the community informed. A presentation in Arabic was conducted before the prototyping phase, as detailed in the Methodology section (page 12), and was delivered during the feedback sessions to facilitate dialogue and incorporate community input.*

**Non-human subjects research using specimens/ animals collected as part of the study, or those housed in archival collections. Examples include archaeology, paleontology, botany and zoology.**

Did the permission you obtained from a local authority to perform the study include an agreement on access to outputs and benefit sharing? This may include procedures to enable fair distribution of the benefits and resources arising from the research performed. Please include any details of Prior Informed Consent and Benefit Sharing Agreements obtained. These may be required by field-specific regulations, for example the Convention on Biological Diversity (CBD) and the associated Nagoya Protocol.

N/A

If the material used in your study was imported, please A) provide the year it was imported and B) indicate whether permits were obtained to import/export the materials used, C) provide details of any permits obtained. If this information is not available, please indicate this.

N/A

If you used archival specimens, please state how the material used in your study was acquired by the institute it is held in and provide details of any permits obtained for the original excavations/ sample collection. If this information is not available, please indicate this.

N/A

How was the potential cultural significance of the materials collected in your study to local communities considered in your research design? Were Indigenous peoples and/or local researchers and institutions involved with archaeological excavations / collection of specimens? If so, please provide a description of their involvement.

N/A

If your manuscript includes photographs of human remains please indicate whether authors obtained permission from descendants or affiliated cultural communities to do so.

N/A
