# Supplementary material for: Developing guidelines for culturally relevant informed consent: an example from Lebanon
Source: PLOS Ment Health. 2025 Apr 17;2(4):e0000174. doi: 10.1371/journal.pmen.0000174 (PMC12798495; doi:10.1371/journal.pmen.0000174)
Supplement: Attachment — Submitted filename: Response to Reviewers.docx [file pmen.0000174.s005.docx]

Dear Reviewers,

We are extremely grateful for your comments and suggestions on our manuscript. We have provided point-by-point responses below, including cross-references to the changes in the revised manuscript (track change version) which are shown as track changes/yellow highlights.

Kind regards. The authors.

| Comments | | Authors’ response |
| --- | --- | --- |
| **Editor’s comments:** | | |
| 1. Please include a complete copy of PLOS’ questionnaire on inclusivity in global research in your revised manuscript. Our policy for research in this area aims to improve transparency in the reporting of research performed outside of researchers’ own country or community. The policy applies to researchers who have travelled to a different country to conduct research, research with Indigenous populations or their lands, and research on cultural artefacts. The questionnaire can also be requested at the journal’s discretion for any other submissions, even if these conditions are not met.  Please find more information on the policy and a link to download a blank copy of the questionnaire here: <https://journals.plos.org/plosmentalhealth/s/best-practices-in-research-reporting>. Please upload a completed version of your questionnaire as Supporting Information when you resubmit your manuscript. | | A completed copy of PLOS' questionnaire on inclusivity in global research has been filled out and submitted (Supporting Information 2), and is also referenced in the methods section (p. 9, paragraph 2) |
| Have the authors made all data underlying the findings in their manuscript fully available (please refer to the Data Availability Statement at the start of the manuscript PDF file)?  The PLOS Data policy requires authors to make all data underlying the findings described in their manuscript fully available without restriction, with rare exception. The data should be provided as part of the manuscript or its supporting information, or deposited to a public repository. For example, in addition to summary statistics, the data points behind means, medians and variance measures should be available. If there are restrictions on publicly sharing data—e.g. participant privacy or use of data from a third party—those must be specified. | | The data availability statement has been added to the ethics section (page 13, paragraph 2). |
| **Reviewer #1’s comments:** | | |
| This is a very important and timely work. The authors aim to improve the informed consent process in research to be more tailored to the needs and perceptions of vulnerable populations with different socio-cultural backgrounds*.* I recommend that the authors replace Figure 1 with a Figure that summarizes their conceptual framework based on the themes extracted from their study. | | Thank you very much for your kind words and suggestions.  We have added a figure (Figure 1) that represents a conceptual framework summarizing key factors influencing the informed consent process in research, based on themes extracted from the study. We reference this figure on page 13, paragraph 2.  This figure has been uploaded as a separate file, as per journal requirements. |
| **Reviewer #2’s comments:** | | |
| **Intro** | Lines 113-117: please provide several references for this statement | Thank you for this and all the other very helpful comments and suggestions.  We have added the following the following references (page 4, paragraph 2):   - Gómez Paciello L, Schippers F, Salina M, Medina A, Gill L, Bordón M, Martinez M, Ortellado G, Aguiar A, Magis-Escurra C. A qualitative assessment of an audio-visual informed consent procedure in a diagnostic trial in penitentiary institutions. SSRN; 2024 Feb 27 - Gesualdo F, Daverio M, Palazzani L, Dimitriou D, Diez Domingo J, Fons-Martinez J, Jackson S, Vignally P, Rizzo C, Tozzi AE. Digital tools in the informed consent process: a systematic review. BMC Med Ethics. Preprint 2024. - Afolabi MO, Bojang K, D'Alessandro U, Imoukhuede EB, Ravinetto RM, Larson HJ, McGrath N, Chandramohan D. Multimedia informed consent tool for a low literacy African research population: development and pilot-testing. J Clin Res Bioeth. 2014;5(3):178. - Jimison HB, Sher PP, Appleyard R, LeVernois Y. The use of multimedia in the informed consent process. J Am Med Inform Assoc. 1998 May-Jun;5(3):245-56. - Reddy KN. Obtaining informed consent for clinical trials in low- and middle-income countries: a systematic review. Baltimore: Johns Hopkins University; 2023. |
|  | Lines 135-137: “Very little research has been conducted regarding how to improve the process of informed consent, especially when working with vulnerable populations such as refugees and those exposed to humanitarian emergencies, to address problems related to human interactions.”  The following reference might be relevant to this statement:  Seagle EE, Dam AJ, Shah PP, Webster JL, Barrett DH, Ortmann LW, Cohen NJ, Marano NN. Research ethics and refugee health: a review of reported considerations and applications in published refugee health literature, 2015-2018. Confl Health. 2020 Jun 20;14:39. doi: 10.1186/s13031-020-00283-z. PMID: 32577125; PMCID: PMC7305588. | Thank you for this suggestion. We have added the suggested reference (page 5, paragraph 2). |
| **Methods** | I suggest that authors should include the Consolidated Criteria for Reporting Qualitative Research (COREQ) and report the extent to which they fulfilled each of the criteria mention in COREQ. | We have now included the COREQ checklist. This has been noted as part of the methodology (page 7, paragraph 2) and this has been uploaded as Supporting Information 1. |
|  | I suggest a table that provides the number of FGDs and IDI including the member composition of each FGD and IDI (including gender make-up). | We have now added this table to the manuscript (Table 2). |
| **Results** | Themes development: It appears that several of the themes can be further unpacked. For example, Theme 1 encompasses “motivations and incentives for participation”. However, some of the quotes suggest an element of coercion, e.g., line 323: “…may feel pressure to participate to maintain.” Considering that the study focused on refugees, I would think coercion and exploitation would be a major sub-theme if not a stand-alone theme.  Considering that there was rich data collection, it would be beneficial to include more representative quotes or examples from participants to provide richer insights into the identified themes. | This suggestion is much appreciated. To further unpack the theme of ‘motivations and incentives for participation,’ particularly in relation to coercion, this topic is now a stand-alone sub-theme as suggested (page 14, paragraph 2). While key quotes that address this aspect have been incorporated, a more detailed quote has also been added to further clarify the researchers' perspective on the matter (starting line 358). We hope that this helps to address the nuance of coercion within the context of the study while still keeping the discussion of the theme concise and focused. |
|  | It would be beneficial to have a table with the themes and subthemes (or codes) if the authors choose to unpack several of the themes. | We have added a table with the themes/sub-themes (Table 3) |
| Discussion | Provide a clear and concise definition of culturally relevant informed consent early in the manuscript, drawing from the literature and the study’s findings. | Added on page 31 (paragraph 2) |
|  | Enhance the discussion of specific cultural practices, norms, or values in Lebanon that influence informed consent, with examples from the study's data to illustrate their impact. | We have added further discussion on this point in Lebanon (page 30, paragraph 2). |
|  | Broaden the discussion to include comparisons with similar studies in different cultural or geographical contexts to highlight universal and specific challenges. | We have added further discussion on this point for different contexts (page 29, paragraph 2). |
|  | Provide more detailed strategies for overcoming language and literacy challenges, possibly with visual examples of tools used. | We have provided more detailed strategies for overcoming language and literacy challenges to the Discussion (page 33, paragraph 2). |
|  | Focus on coercion: While the authors discuss power dynamics, the discussion is not explicitly linked to coercion or the risk of undue influence. Power dynamics are framed more as a structural issue than as a direct factor in decision-making about research participation.  As implied by the authors, refugees often live in precarious socio-economic conditions, relying on aid and services provided by NGOs or governments. This dependency can create a perception that participation in research is obligatory or that declining could jeopardize access to essential resources or future support.  Even when researchers do not intend coercion, refugees may feel compelled to participate due to implicit pressures, such as:  · A sense of obligation to reciprocate for humanitarian assistance.  · Fear of repercussions for non-participation.  · Misunderstanding the voluntary nature of research participation due to language barriers or power imbalances.  Finally, refugees are often over-researched populations, which can exacerbate feelings of exploitation or distrust. This history compounds the risk of coercion, as individuals may feel powerless to decline participation. | Thank you for these important points. We have added them to the Discussion (particularly page 32, paragraphs 3 and 4). |
| Finally, would it be useful to develop a general check list for culturally relevant informed consent? | | Thank you for this suggestion. This has been added as Supporting Information 3, and we have made a note of this in the manuscript (page 29, paragraph 1). |
|  | | Thank you again for all the very helpful suggestions. |
